# Chromatin immunoprecipitation improvements for the processing of small frozen pieces of adipose tissue

**DOI:** 10.1371/journal.pone.0192314

**Published:** 2018-02-14

**Authors:** Daniel Castellano-Castillo, Pierre-Damien Denechaud, Isabel Moreno-Indias, Francisco Tinahones, Lluis Fajas, María Isabel Queipo-Ortuño, Fernando Cardona

**Affiliations:** 1 Unidad de Gestión Clínica de Endocrinología y Nutrición del Hospital Virgen de la Victoria, Instituto de Investigación Biomédica de Málaga (IBIMA), Universidad de Málaga, Málaga, Spain; 2 Centro de Investigación Biomédica en Red de Fisiopatología de la Obesidad y la Nutrición, CIBERobn, Madrid, Spain; 3 Center for Integrative Genomics, University of Lausanne, Lausanne, Switzerland; 4 Department of Physiology, University of Lausanne, Lausanne, Switzerland; 5 Institut des Maladies Métaboliques et Cardiovasculaires, Inserm UMR 1048, Toulouse, France; Tokyo Daigaku, JAPAN

## Abstract

Chromatin immunoprecipitation (ChIP) has gained importance to identify links between the genome and the proteome. Adipose tissue has emerged as an active tissue, which secretes a wide range of molecules that have been related to metabolic and obesity-related disorders, such as diabetes, cardiovascular failure, metabolic syndrome, or cancer. In turn, epigenetics has raised the importance in discerning the possible relationship between metabolic disorders, lifestyle and environment. However, ChIP application in human adipose tissue is limited by several factors, such as sample size, frozen sample availability, high lipid content and cellular composition of the tissue. Here, we optimize the standard protocol of ChIP for small pieces of frozen human adipose tissue. In addition, we test ChIP for the histone mark H3K4m3, which is related to active promoters, and validate the performance of the ChIP by analyzing gene promoters for factors usually studied in adipose tissue using qPCR. Our improvements result in a higher performance in chromatin shearing and DNA recovery of adipocytes from the tissue, which may be useful for ChIP-qPCR or ChIP-seq analysis.

## Introduction

Epigenetic regulation, generally histone marks and DNA methylation, is a regulatory process which depends on stochastic and environmental stimuli; while DNA methylation is a process usually associated with gene repression that, in mammals, occurs mainly at cytosine residues located within CpG islands, histone marks can exert an activation or repression effect depending on the type of chemical modification and on which residue is modified [1,2].

**Chromatin immunoprecipitation (ChIP)** has raised importance in discerning protein-DNA interactions *in vivo* [3], allowing study of epigenetic markers and transcription factor DNA binding and therefore, gene regulation [4–6]. The downstream usage of DNA usually involves qPCR, DNA arrays or sequencing [7].

Adipose tissue (AT) has traditionally been regarded as merely a storage tissue entrusted with storing the surplus energy in the organism. Nevertheless, AT is nowadays considered an endocrine organ, which can be an important and active tissue that secretes molecules that can be implicated in systemic homeostasis and in several diseases [8–11]. In the last years, the epigenetic implication of AT in the etiology of comorbidities associated with malfunction in this tissue has been studied [12–14]. However, most epigenetic studies have been performed focusing on the role of DNA methylation, while few studies have been carried out on the implication of the histone landscape.

This fact is supported by the nature of AT. AT is composed of a multitude of cell types, such as adipocytes and stromal vascular fraction [15], which makes ChIP applicability difficult. The high lipid composition complicates the extraction of DNA and may lead to major DNA recovery from stromal cells. Moreover, the use of this technique has been limited by the restricted quantity of frozen tissues. Therefore, most studies have focused on AT mesenchymal cells, fresh mouse AT, and a human ChIP protocol has only been designed using huge amounts of fresh tissue [16–18].

In this study we propose a series of improvements to the regular ChIP protocol in order to make the ChIP technique applicable for the study of small frozen pieces of AT, which could facilitate the study of epigenetic regulation of human AT biobanks and thus enhance the knowledge and role of histone in AT-associated disorders.

## Methods

### Human adipose tissue collection

Study subjects (12 men and 27 women, aged 46.18±11.09 years), were recruited during 2012–2014 from patients that had undergone laparoscopic surgery for elective cholecystectomy, hiatal-hernia surgery, or bariatric surgery. AT was collected and washed in PBS, after which it was frozen with liquid nitrogen and then stored at -80°C.

The study was conducted in accordance with the guidelines laid down in the Declaration of Helsinki. All participants gave their written informed consent and the study was reviewed and approved by the Ethics and Research Committee of Virgen de la Victoria Hospital.

### Tissue fixation and homogenization

Due to the limitations concerning human tissue samples, we chose to test ChIP on 100 mg of frozen mouse AT as a starting material to set up the protocol.

First, we established the optimum homogenization method according to the most widely used procedure with other biological tissues for ChIP: mortar and pestle, to grind the samples with liquid nitrogen in order to preserve the tissue and facilitate the extraction, was compared with two other homogenization methods using either a Dounce homogenizer or a Ultraturrax homogenizer (Ika, Sigma-Aldrich).

In the standard method (Fig 1.1) the tissue was first homogenized with the help of liquid nitrogen and a pestle and mortar. Once homogenized, the tissue was fixed using 5 ml of two different concentrations, 1% or 0.5% of formaldehyde solution in PBS for 5 minutes at room temperature (RT). The fixation step was stopped adding glycine to a final concentration of 0.125 mM and incubating for 5 minutes at RT and shaking. The sample was centrifuged at 1500 rpm and 4°C, and both the upper adipocyte fraction and the pellet were collected by removing the intermediate liquid phase with a glass pipette. After this, the sample was washed twice in cold PBS plus proteinase inhibitor cocktail (PIC) (Thermofisher) and centrifuged as in the previous step. Then, 5 ml of cell lysis buffer (5 mM PIPES pH8, 85 mM KCl, 1% NP40) plus PIC were added and the sample was incubated for 30 minutes in ice while vortexing every 3 minutes. The sample was centrifuged at 2870×g for 10 minutes at 4°C to precipitate the cell nuclei. The supernatant was removed and 300 μl of cold nuclei lysis buffer (50 mM Tris HCl pH 8,1, 10 mM EDTA, 1% SDS) plus PIC were added and it was transferred to a 1.5 ml polypropylene tube. After incubating for 1 hour on ice the sample was ready for the chromatin shear step.

**Fig 1:**
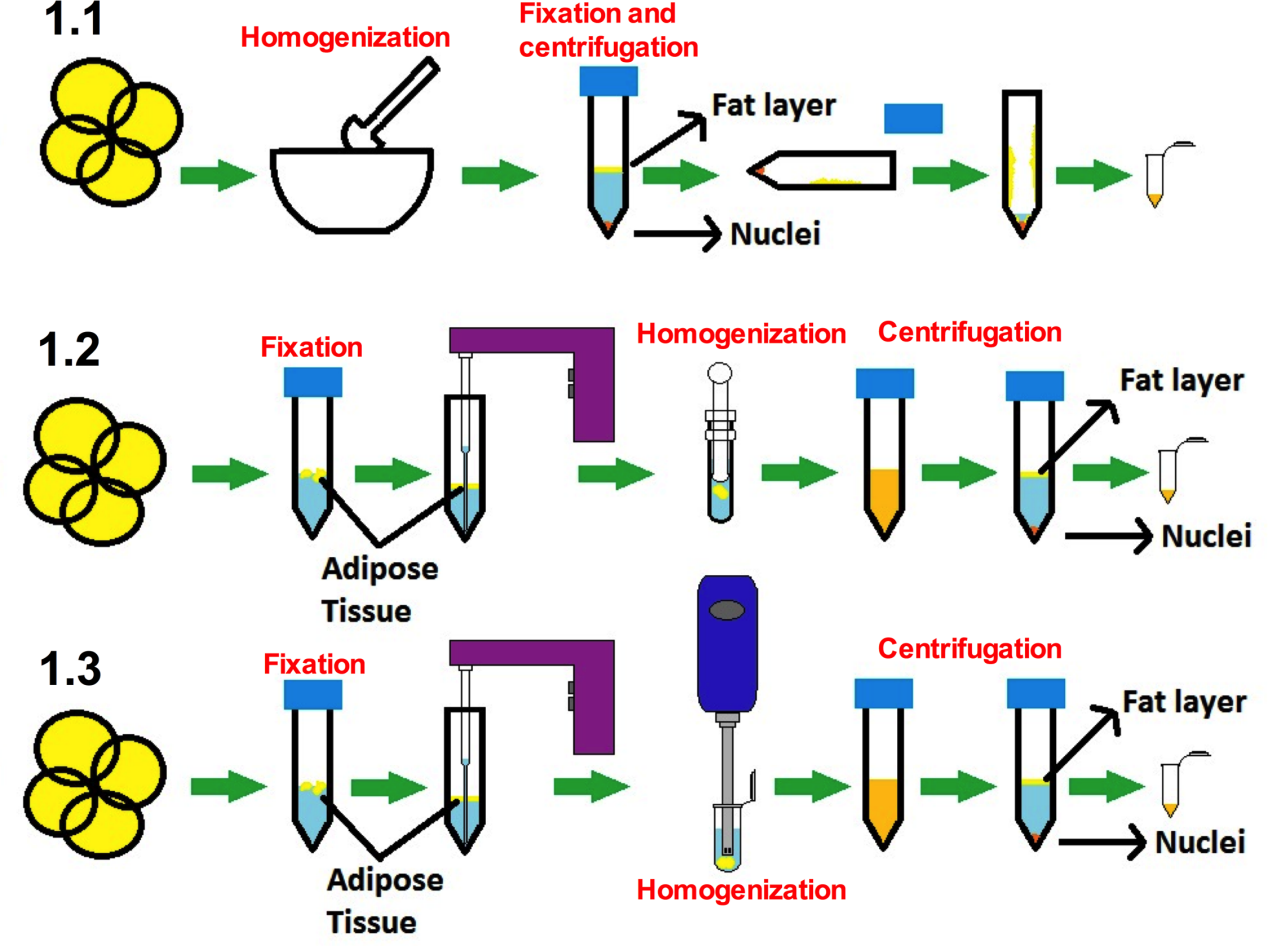
The chart shows the three different workflows performed, using the pestle and mortar (1.1), the Dounce homogenizer (1.2) or the Ultraturrax homogenizer (1.3). In (1.1), the homogenization step was performed using liquid nitrogen, after which it was fixed. After this, the nuclei were pelleted and nucleus lysis buffer was added. Once incubated, the sample was sheared and the chromatin fragmentation and recovery were checked. In the other two alternative methods proposed (1.2 and 1.3), the tissue was cut in small pieces (3 mm) and the fixation step was performed prior to the homogenization. Once homogenized, the nuclei were recovered by centrifugation, nucleus lysis buffer was added and the sample was sheared and the chromatin fragmentation and recovery were checked.

We also tested two other methods of homogenization, the Dounce homogenizer (Fig 1.2) that has been previously tested in fresh AT [18], and the Ultraturrax homogenizer (Fig 1.3), which has been demonstrated to be useful in the extraction of RNA from frozen AT [19]. In both cases, the fixation step was performed prior to the homogenization step using 5 ml of fixing solution (PBS + formaldehyde 1% or PBS + formaldehyde 0.5%) over 100 mg of AT (previously chopped into small pieces of about 3 mm) for 5 minutes at RT. Then, the fixation was stopped adding glycine to a final concentration of 0.125 mM and incubating for 5 minutes at RT and shaking, after which it was centrifuged at 1500 rpm and 4°C. The liquid phase was then removed with a glass pipette. The next steps were performed at 4°C. The tissue was washed twice in 5 ml of cold PBS plus PIC, then centrifuged at 1500 rpm and 4°C and the liquid phase discarded with the help of a glass pipette. The tissue was then homogenized, either with the Dounce (20 strokes with the loose pestle and 20 strokes with the tight pestle) or the Ultraturrax homogenizer (two pulses of 6 seconds each with a resting period between them) in 5 ml of cold cell lysis buffer plus PIC and incubated on ice for 30 minutes with vortex every 3 minutes. After the incubation, the sample was centrifuged at 2870×g in order to precipitate the nuclei fraction. The supernatant liquid phase was discarded by inversion and the nuclei pellet was suspended in 300 μl of nuclei lysis buffer plus PIC and transferred to a 1.5 ml polypropylene tube and then incubated on ice for 1h (it is important to prevent the fat on the walls of the tube falling down to the nuclei pellet). After the incubation, the sample was prepared for the chromatin shear step.

### Chromatin shear and DNA de-crosslinking step

For the sonication a Bioruptor UCD-300 (Diagenode) was used. The samples were sheared at high power for 20, 30 or 40 cycles of 30 seconds ON and 30 seconds OFF. After this, 50 μl (Pre-Input) of sample was taken and the DNA de-crosslinked and purified in order to check the chromatin shear performance.

The de-crosslinking step was carried out using two methods, the standard method based on incubating the sample at a moderate temperature for a long period of time and another protocol based on submitting the sample to a high temperature for a short period of time. In the standard protocol the sample was incubated at 65°C for 5 h (instead of 5 h the sample can be incubated overnight). After this incubation, the samples were treated with proteinase K proteolysis in order to facilitate the DNA release. Then, TNESKx5 (50 mM Tris-HCl pH 7.5, 500 mM NaCl, 5 mM EDTA, 5% SDS) plus proteinase K (PK) (New England Biolabs) were added to the sample and incubated at 55°C for 1h. In the alternative method tested, 10% of chelex-100 (Sigma-Aldrich) was added to the sample after which it was incubated at 100°C for 10 minutes.

Once the chromatin was de-crosslinked, the DNA was purified using the MinElute PCR Purification Kit (Qiagen) according to the manufacturer’s procedure, and the chromatin shearing performance was tested in 2% agarose gel.

### Chromatin immunoprecipitation assay

First of all, the magnetic balls (Dynabeads Protein G, Thermofisher) used in the immunoprecipitation (IP) were pre-cleaned in order to minimize unspecific chromatin binding. Thus, the magnetic balls were diluted in pre-cold IP dilution buffer (0.01% SDS, 1.1% triton 100x, 1.2 mM EDTA, 16 mM Tris-HCl pH 8.1, 167 mM NaCl) plus PIC at a magnetic balls:buffer proportion of 3:100. Furthermore, sonicated salmon sperm (UltraPure™ Salmon Sperm DNA Solution, Thermofisher) and BSA (Bovine serum albumin, Thermofisher) to a final concentration of 1 mg/ml were added (0.5 mg of total salmon sperm and BSA). The magnetic balls were incubated for 1h at 4°C and in rotation. After this incubation period we removed the supernatant with the help of a DynaMag™-2 magnet (Thermofisher).

Afterwards, the magnetic balls were incubated for 30 minutes at 4°C in rotation with 1.5 μg of anti-H3K4m3 (ab8580, abcam) in 600 μl of IP dilution buffer. As IP control we used rabbit anti-IgG (ab171870, abcam). The liquid phase was then removed as in the previous step.

After the antibody was linked to the balls, 5 μg of chromatin was used to perform the IP step. Thus, up to 200 μl of chromatin was added to complete a total volume of 2 ml diluted in IP dilution buffer for each IP, after which it was incubated at 4°C and in rotation overnight.

After the incubation, the sample was submitted to several washing steps, from low salt content to high salt content buffers. First the liquid phase was removed and 700 μl of pre-cold wash buffer A (20 mM Tris-HCl pH 8.1, 2 mM EDTA, 150 mM NaCl, 1% Triton x100, 0.1% SDS) plus PIC were added. Then, the content was transferred to a low DNA binding capacity 1.5 ml microcentrifuge tube (Eppendorf® LoBind microcentrifuge tubes). After this, the liquid phase was removed using a DynaMag™-2 magnet. Washing steps using wash buffer B (20 mM Tris-HCl pH 8.1, 2 mM EDTA, 500 mM NaCl, 1% Triton x100, 0.1% SDS), LiCl buffer TP3 (10 mM Tris-HCl pH 8.1, 1mM EDTA, 1% Deoxycholate, 1% NP-40, 0.25 M LiCl) and TE buffer (10mM Tris-HCl pH 8.1, 1mM EDTA) were performed in the same way as the washing step with buffer A. The chromatin was then eluted using 60 μl of elution buffer (0.1 M NaHCO3, 1% SDS) and incubating the sample at 37°C for 10 minutes. Afterwards, the elution was transferred to a new low DNA binding capacity microcentrifuge tube. The elution step was repeated using another 60 μl after which a total of 120 μl of chromatin was recovered.

In order to normalize the results an Input (chromatin without immunoprecipitation) for each IP was established. Thus, we took 20% of the chromatin used for the IP as Input, which was diluted in elution buffer up to a total of 120 μl. The immunoprecipitated chromatin and the Input were de-crosslinked by submitting the sample to 65°C for 4 h. After the incubation, 3 μl of NaCl 5M, 10 μl of TRIS 1M, 1 μl of RNAse 1mg/ml and 1 μl of PK 1mg/ml were added and the sample was incubated at 55°C for 1h. The DNA was purified using the MinElute PCR Purification Kit (Qiagen) in accordance with the manufacturer’s instructions. The DNA was eluted twice in 25 μl of water, until a total volume of 100 μl was obtained.

### qPCR quantification

In order to test the performance of the ChIP, the promoter for several genes usually studied in AT was quantified by qPCR. The primer sequences used are displayed in Table 1 for mice and Table 2 for humans. As control regions we used a sequence 30 kb before PPARG TSS (Transcription Start Site) in mice (PPARG Out) and a sequence 35 kb before SCD TSS (SCD Out) in humans (Tables 1 and 2, respectively). For the qPCR quantification 5 μl of eluted DNA was used for each reaction. The quantification was carried out using FastStart Universal SYBR Green Master (Rox) (Roche) and its corresponding standard curve; 20% input was used to normalize the result for each gene. The delta CT value was calculated as the subtraction of the IP Ct with the Input Ct. The ChIP enrichment was calculated by comparing the CT values obtained from the standard curves. Standard curves were constructed for both mice and humans using four-fold dilutions of Input samples (20, 5, 1.25 and 0.375% of the Input).

**Table 1:** The table shows the primer pairs used for each gene for mice. The primer pairs included a control region of the PPARG gene to evaluate the correct performance of the IP. Abbreviations: Transcription start site (TSS); Nucleotides (nt).

Gene	Primer Sequence	nt from the TSS (pb)
**LEP**	Sense: GCAGACTTGAGATGGTTAGG Antisense: CTTCAGGAAGGCGGAAAG	+299 to +378
**LPL**	Sense: GTGTCAGACTCTCGATTTCTC Antisense: GTAGGGCAAGTCAACCTTTA	-6 to 77
**SREBP2**	Sense: CGATGACGCACCATCAC Antisense: TTGTTGTCAATGGGACCAG	-176 to -103
**SCD1**	Sense: TCTGGAAGCTCACCTCTT Antisense: AAGTCCACGCTCGATCT	+335 to +246
**PPARG**	Sense: CTGAGGAGAAGTCACACTCT Antisense: TGTCACACAGTCCTGTCA	+38 to +129
**IL6**	Sense: GGATGTCTGTAGCTCATTCTG Antisense: GGAACTGCCTTCACTTACTT	-34 to +68
**TNFa**	Sense: AGAAGAGGCTGAGACATAGG Antisense: GACACCATGAGCACAGAAA	+266 to 153
**PPARG out**	Sense: CTTCCGTTCCTCACTCTAATC Antisense: GACTCAGGATGGCTAACTATAAC	-31.7 kb from PPARG TSS

**Table 2:** The table shows the primer pairs used for each gene for humans. The primer pairs included a control region of the SCD gene to evaluate the correct performance of the IP. Abbreviations: Transcription start site (TSS); Nucleotides (nt).

Gene	Primer Sequence	nt from the TSS (pb)
**LEP**	Sense: GCTGAGATGCATTGGAAATTG Antisense: CCCAACTTTATCTCCTTCAGAC	+211 to +306
**LPL**	Sense: GCATATTTCCAGTCACATAAGC Antisense: CTAGAAGTGGGCAGCTTTC	+140 to +235
**SREBP2**	Sense: AGGCGGAGAAGGTTAAGA Antisense: CGATCAGCAGCTCAGATTT	-244 to -143
**SCD**	Sense: GAGAAGCTGAGAAGGAGAAAC Antisense: TTGGCCGAAGGGAATTTG	-100 to +50
**PPARG**	Sense: AAACTTCGGATCCCTCCT Antisense: GCTACCTGGTGTCGTTTG	-150 to -250
**IL6**	Sense: CCTGCATTAGGAGGTCTTTG Antisense: CTGACACCAGCAAAGGATAA	+572 to +675
**TNFa**	Sense: GTAGCCCATGTTGTAGGTAAG Antisense: CAAGTTCTGCCTACCATCAG	-296 to -194
**SCD out**	Sense: GCCACAGGATATGAGCATTAG Antisense: GGTAAAGAAGTGGAGGAGTAGA	-35kb from SCD TSS

## Results and discussion

Here, we have shown for the first time a complete ChIP method for the study of small pieces of frozen AT. We have introduced crucial changes to the standard ChIP protocol, improving the homogenization, fixation and de-crosslinking steps, allowing enough immunoprecipited material to be obtained to perform further steps, as we demonstrated by testing H3K4me3 modifications. Thus, we have shown that the use of only 100 mg of frozen AT is enough for ChIP tests, which will help to advance knowledge about epigenetic marks of AT and their significance for metabolic homeostasis.

**Fig 2:**
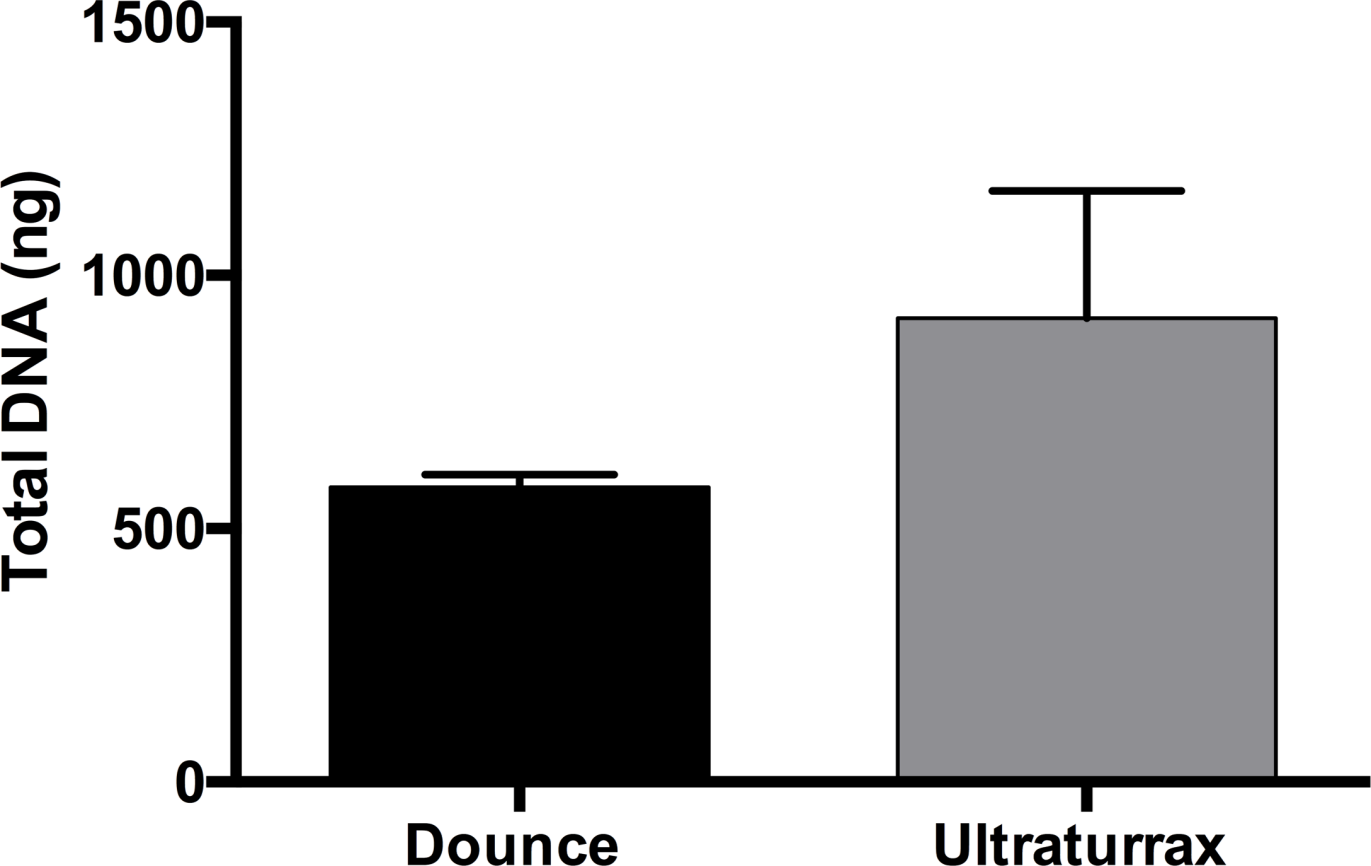
The chart shows the different efficiency in total DNA recovery between the Dounce and the Ultraturrax homogenizer. 100 mg of frozen adipose tissue was fixed in 1% paraformaldehyde, homogenized either using the Dounce or Ultraturrax homogenizer and sheared for 40 cycles (30 seconds ON and 30 seconds OFF). A sample of 50 μl of the homogenized material was then taken, and the chromatin was de-crosslinked using the fast Chelex-100 method. Total DNA was extracted and quantified by nanodrop. Data are given as means with error bars. Abbreviations: Dounce, Dounce homogenization method; Ultraturrax, ultraturrax homogenization method. (n = 6).

The high lipid content of the AT makes the fixation and subsequent steps difficult to work with. Adipocytes float in the upper layer [20] due to their lipid content (Fig 1.1), which leads to a high loss of tissue in the processing. Thus, standard homogenization methods (mortar and pestle) were not able to extract a proper quantity of DNA, showing a very low performance. In this method, a high quantity of tissue remained stuck to the surfaces of the pestle and mortar that resulted in a high tissue loss, a very low nuclei recovery and no chromatin harvest. We therefore performed two other alternative methods where the fixation and washing of the tissue were prior to the homogenization step. This allowed better tissue manipulation, indeed avoiding loss of tissue. We compared the dounce homogenizer (Fig 1.2) with the ultraturrax homogenizer (Fig 1.3). The use of the ultraturrax homogenizer results in a higher total DNA recovery after the purification step (Fig 2).

**Fig 3:**
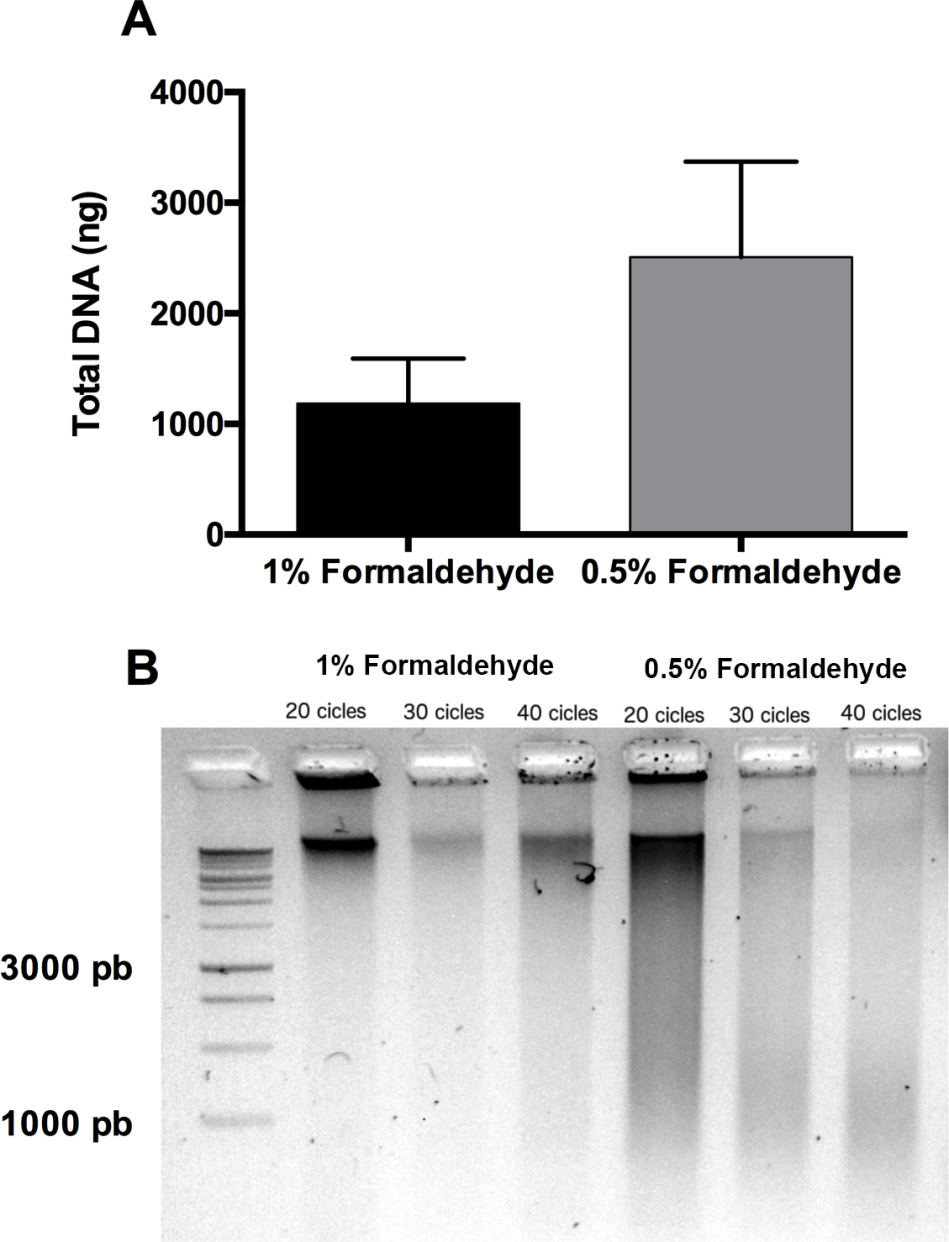
100 mg of frozen adipose tissue was fixed in either 1% or 0.5% of paraformaldehyde and then homogenized using the Ultraturrax method. It was then sheared for 40 cycles (30 seconds ON and 30 seconds OFF), after which a sample of 50 μl of the homogenized material was taken, and the chromatin was de-crosslinked using the fast Chelex-100 method. Total DNA was extracted and quantified by nanodrop. Fixation at 0.5% presents higher levels of DNA recovery (A) after DNA purification and a better chromatin shear tested by electrophoresis in 2% agarose gel. (B) Comparison of the use of PBS+1% or PBS+0.5% formaldehyde in the performance of DNA recovery after de-crosslinking and purifying the DNA. (n = 6). Data are given as means with error bars.

Once the optimal homogenization method was established, a proper fixation method for AT was found. Concentration of formaldehyde to crosslinking DNA/protein is an important step, which can affect the shearing of the DNA and, consequently, the performance of the IP and the recovery of DNA [3]. For this reason, we tried two different formaldehyde concentrations in order to improve DNA recovery: the classical concentration of 1% formaldehyde [21] was compared to a lower concentration of 0.5% formaldehyde. In addition, we also tried different incubation times (10, 8, 5 minutes) and temperatures (RT or 37°) to fix the sample, although no good results were obtained for incubation times longer than 5 minutes and temperatures higher than RT (data not shown). 5 ml of each fixation solution were used to carry out the fixation step for 5 minutes at RT and shaking. Furthermore, the sonication step is highly variable depending on the sonicator and there is even moderate variation between different devices for the same technology. Indeed, it is recommendable to set up the proper shearing method not only for each kind of tissue but also for each device. At the same time, we also determined the sonication time to properly shear the DNA using a Bioruptor sonicator after tissue fixation. We tested 20, 30 and 40 cycles of 30 seconds ON / 30 seconds OFF at high power. We obtained better results using a low concentration of 0.5% formaldehyde together with a number of 40 cycles for DNA recovery (Fig 3A and 3B). However, 1% formaldehyde hindered shearing of the chromatin, independently of the number of sonication cycles.

**Fig 4:**
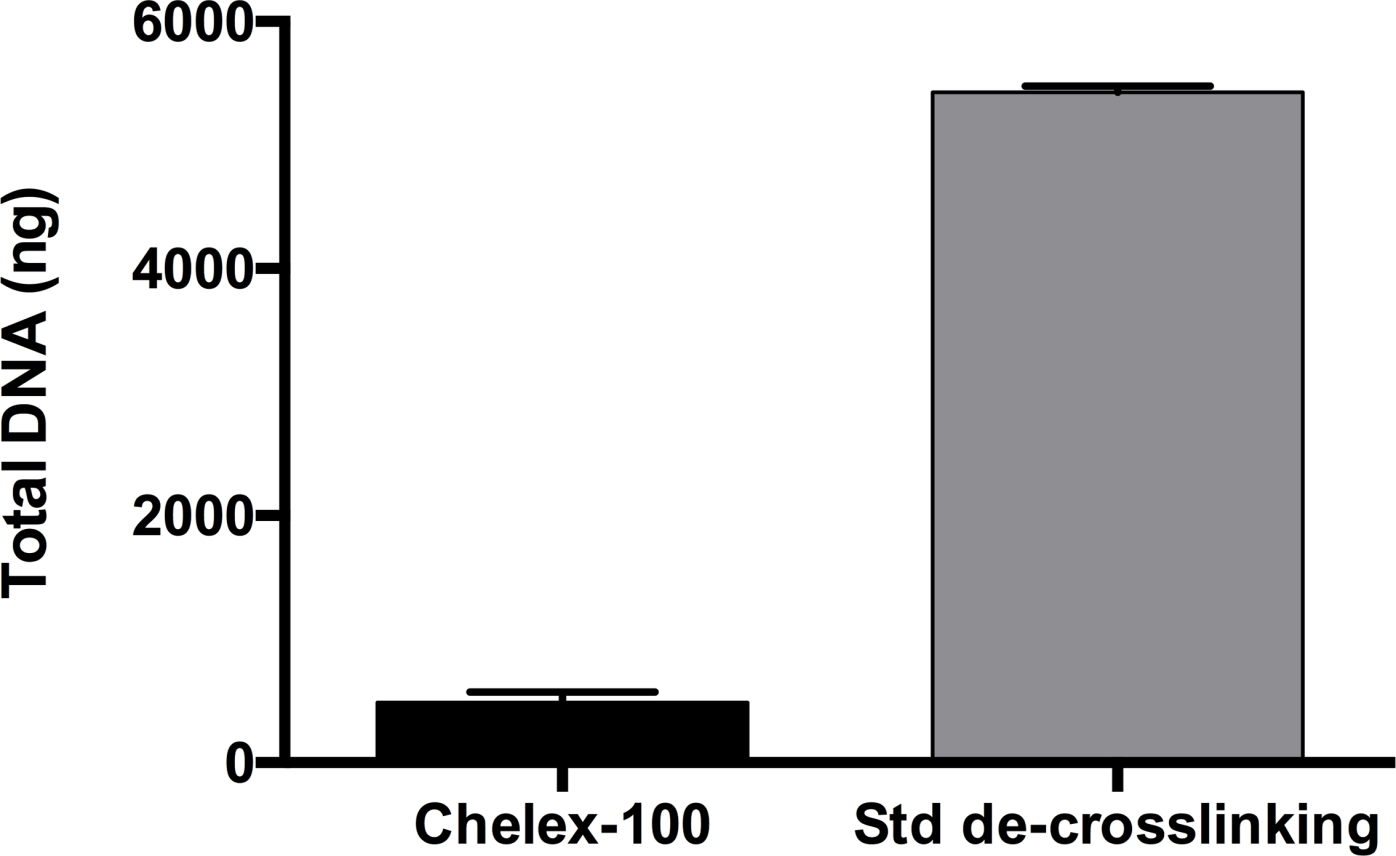
100 mg of frozen adipose tissue was fixed in 0.5% paraformaldehyde and then homogenized using the Ultraturrax method. It was then sheared for 40 cycles (30 seconds ON and 30 seconds OFF), after which a sample of 50 μl of the homogenized material was taken, and the chromatin was de-crosslinked using either the fast Chelex-100 method or a moderate temperature for 5 hours plus PK treatment. Fig 4 shows the de-crosslinking step at a moderate temperature for 5 hours and then a PK step improves the quantity of DNA with respect to the method based on the use of Chelex-100 (n = 6) Total DNA was extracted and quantified by nanodrop. Data are given as means with error bars.

Finally, due to the small pieces and nature of AT itself, added to the fact that it was frozen, the de-crosslinking and DNA recovery steps may be determinant for the success of the IP and downstream procedures. Two methods for chromatin de-crosslinking were tested: the standard method, which consists of incubating the chromatin at 65°C for 5 hours followed by proteinase K (PK) treatment at 55° for 1 hour [16]; and a faster method in which chromatin is heated to 100°C during a shorter period of time of 10 minutes with 10% chelex-100 to protect the DNA [22]. Once the DNA was purified, the data revealed a higher performance for the standard method, in which the chromatin is submitted to a moderate temperature for a long time (Fig 4).

Therefore, after testing different steps during the regular ChIP protocol, several changes have been introduced in order to match the method to small frozen AT samples. The high lipid content in AT hinders tissue manipulation, DNA extraction and even nucleus release and nucleus breakdown [23]. This has led to the development of specialized extraction kits for AT, for example for RNA extraction [24]. Thus, based on our data in AT manipulation (data not shown), we decided to increase the proportion of buffer with respect to the sample quantity compared to regular procedures for the following steps: fixation, washes after fixation, cell lysis and release of nuclei. This allowed us to deal with the high lipid content, avoiding a very thick cell lysate which could hinder nucleus release. Furthermore, this allowed recovery of a cleaner nucleus pellet, improving the sonication and chromatin release. In these steps, we recommend the use of glass pipettes to remove the liquid discarded in each step since the high lipìd content of AT can become stuck to plastic surfaces, hindering manipulation and leading to tissue loss. Moreover, we determined use of the ultraturrax homogenizer as the best method for homogenization, a fixing solution of PBS+0.5% formaldehyde at RT and the standard de-crosslinking method as the most suitable procedures for small pieces of frozen AT. Up to now, the use of ChIP for AT has been limited to big amounts of tissue [18], and especially to mouse AT [17] where the conditions are less limiting. Thus, the improvements shown in this work could help researchers study the proteome-DNA interaction in human AT, which is stored frozen in large tissue banks.

Once the best procedure was established, we applied the method to 100 mg of frozen samples of human AT. The yield of the method after the IP resulted in an average of almost 100 ng of DNA, enough to perform a posterior high throughput sequencing thanks to the high resolution of the latest next generation sequencing methods [25–27]. On the other hand, in order to improve the performance and DNA recovery, we encourage others to perform ChIP experiments in small rounds of samples.

**Fig 5:**
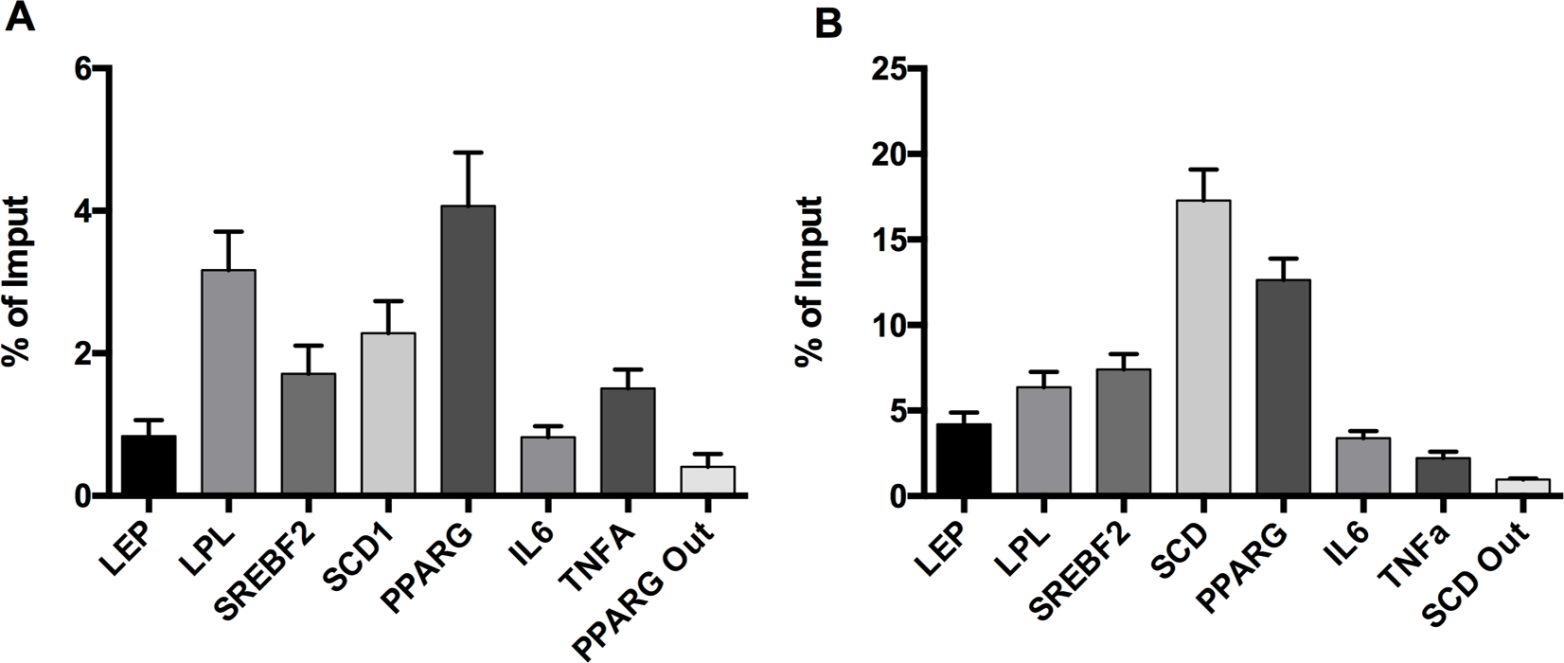
The chart shows the amplification of promoter zones for genes usually expressed in adipose tissue in mice (n = 10) (A) and humans (n = 39) (B), showing that DNA enrichment was successful since a higher quantification was found at the promoter of these active genes but not in the control sequence zones. Data are given as means with error bars. Abbreviations: LPL, Lipoprotein lipase; LEP, Leptin; SREBF2, Sterol regulatory element binding transcription factor 2; SCD, Stearoyl-CoA desaturase; SCD1, Stearoyl-CoA desaturase 1; PPARG, Peroxisome proliferator activated receptor gamma; IL6, Interleukin 6; TNF, tumor necrosis factor; PPARG Out, Sequence 30 kb before PPARG TSS; SCD Out, Sequence 35 kb before SCD TSS.

Although we have provided an improved method to work with small frozen pieces of AT, we needed to confirm the correct assessment of the IP. We validated our ChIP protocol in mouse and human AT by testing H3K4me3 modifications, a mark of active promoter regions [2]. By qPCR we identified H3K4me3 enrichment on several promoters of genes usually expressed in AT, such as PPARG, SCD, LPL, LEP, SREBF2, as well as a sequence 30 kb before PPARG TSS (Transcription Start Site) in mice (PPARG Out) and a sequence 35 kb before SCD TSS (SCD Out) for humans, both as control regions. We obtained a high percentage of enrichment in both mice and humans (Fig 5A and 5B respectively) for genes usually expressed in AT, like SCD, PPARG, LPL or SREBF2, while control regions presented residual expressions. The presence of H3K4m3 at these promoters has already been demonstrated in several tissues and cell lines (ENCODE project) [28], but to the best of our knowledge, no results are available in white AT. Nevertheless, these gene expressions are usually assessed in AT [29–31], which could agree with the high percentage of DNA immunoprecipitation observed in these promoter genes for H3K4m3, a histone associated with active genes [32].

We have shown a novel, improved and reproducible ChIP method for small pieces of frozen AT with several critical steps. It is recommended to fix the whole tissue before mincing it in 5 ml of PBS + 0.5% formaldehyde, after which we recommend several washing and cell lysis steps with abundant cold PBS+PIC. Furthermore, the use of the ultraturrax homogenizer improves nucleus pellet recovery. Afterwards, optimization of the chromatin shear is a key step to success in the following IP. Finally, we recommend de-crosslinking the chromatin under a moderate temperature for 5 hours, adding PK in order to degrade the protein fraction and improve the DNA recovery.
